# *Clonorchis sinensis* omega-class glutathione transferases play major roles in the protection of the reproductive system during maturation and the response to oxidative stress

**DOI:** 10.1186/s13071-016-1622-2

**Published:** 2016-06-13

**Authors:** Jeong-Geun Kim, Chun-Seob Ahn, Seon-Hee Kim, Young-An Bae, Na-Young Kwon, Insug Kang, Hyun-Jong Yang, Woon-Mok Sohn, Yoon Kong

**Affiliations:** Department of Molecular Parasitology, Sungkyunkwan University School of Medicine, 2066 Seobu-ro, Jangan-gu, Suwon, 16419 Korea; Department of Microbiology, Graduate School of Medicine, Gachon University, Incheon, Korea; Department of Molecular Biology and Biochemistry, School of Medicine, Kyung Hee University, Seoul, Korea; Department of Parasitology, Ewha Womans University, School of Medicine, Seoul, Korea; Department of Parasitology and Tropical Medicine, Institute of Health Sciences, Gyeongsang National University College of Medicine, Jinju, Korea

**Keywords:** *Clonorchis sinensis*, Glutathione transferase (GST), Omega-class GST, Sexual maturation, Reproductive system, Oxidative stress

## Abstract

**Background:**

*Clonorchis sinensis* causes a major food-borne helminthic infection. This species locates in mammalian hepatobiliary ducts, where oxidative stressors and hydrophobic substances are profuse. To adapt to the hostile micromilieu and to ensure its long-term survival, the parasite continuously produces a diverse repertoire of antioxidant enzymes including several species of glutathione transferases (GSTs). Helminth GSTs play pertinent roles during sequestration of harmful xenobiotics since most helminths lack the cytochrome P-450 detoxifying enzyme.

**Methods:**

We isolated and analyzed the biochemical properties of two omega-class GSTs of *C. sinensis* (CsGSTo1 and CsGSTo2). We observed spatiotemporal expression patterns in accordance with the maturation of the worm’s reproductive system. Possible biological protective roles of CsGSTos in these organs under oxidative stress were investigated.

**Results:**

The full-length cDNAs of *CsGSTo1* and *2* constituted 965 bp and 1,061 bp with open reading frames of 737 bp (246 amino acids) and 669 bp (223 amino acids). They harbored characteristic N-terminal thioredoxin-like and C-terminal α-helical domains. A cysteine residue, which constituted omega-class specific active site, and the glutathione-binding amino acids, were recognized in appropriate positions. They shared 44 % sequence identity with each other and 14.8–44.8 % with orthologues/homologues from other organisms. Bacterially expressed recombinant proteins (rCsGSTo1 and 2) exhibited dehydroascorbate reductase (DHAR) and thioltransferase activities. DHAR activity was higher than thioltransferase activity. They showed weak canonical GST activity toward 1-chloro-2,4-dinitrobenzene. *S*-hexylglutathione potently and competitively inhibited the active-site at nanomolar concentrations (0.63 and 0.58 nM for rCsGSTo1 and 2). Interestingly, rCsGSTos exhibited high enzyme activity toward mu- and theta-class GST specific substrate, 4-nitrobenzyl chloride. Expression of CsGSTo transcripts and proteins increased beginning in 2-week-old juveniles and reached their highest levels in 4-week-old adults. The proteins were mainly expressed in the elements of the reproductive system, such as vitelline follicles, testes, seminal receptacle, sperm and eggs. Oxidative stressors induced upregulated expression of CsGSTos in these organs. Regardless of oxidative stresses, CsGSTos continued to be highly expressed in eggs. CsGSTo1 or 2 overexpressing bacteria demonstrated high resistance under oxidative killing.

**Conclusions:**

CsGSTos might be critically involved in protection of the reproductive system during maturation of *C. sinensis* worms and in response to oxidative conditions, thereby contributing to maintenance of parasite fecundity.

**Electronic supplementary material:**

The online version of this article (doi:10.1186/s13071-016-1622-2) contains supplementary material, which is available to authorized users.

## Background

*Clonorchis sinensis* causes one of the major fish-borne-zoonotic trematodiases. It is prevalent in several countries of Asia, especially where aquaculture systems associated with paddy field are widely used [1]. Approximately 35 million people are infected and another 600 million people are at risk worldwide [2]. Humans are infected by eating raw/undercooked freshwater fish infected with metacercariae. Light infections usually are asymptomatic. However, chronic cumulative infections invoke several hepatobiliary symptoms including cholecystitis, obstructive jaundice, cholangitis and ascites [3]. Pathological alterations like adenomatous hyperplasia and/or dysplasia of the biliary epithelium, mucin-secreting metaplasia and ductal dilatation with fibrosis are frequently observed in those patients [4]. Epidemiological evidence indicates that approximately 10 % of cholangiocarcinoma is associated with chronic *C. sinensis* infections [5, 6]. Long-standing inflammations accompanied by clonorchiasis might result in oxidative damage of the biliary ductal epithelium and malignant transformation. *C. sinensis* is classified as a Group 1 biocarcinogen [7].

*Clonorchis sinensis* survives more than ten years within the biliary lumen, where free oxygen radicals generated by lipid peroxidation and several hydrophobic substances derived from liver metabolism prevail [8]. In order to adapt to the hostile micromilieu, *C. sinensis* continuously produces diverse antioxidant enzymes, among which several species of glutathione transferases (GSTs: E.C. 2.5.1.18) are the major components [9, 10]. At least eight proteoforms of mu- and sigma-class GST isozymes have been described. Some are intimately involved in protection of the worm during oxidative stress as well as in neutralization of cytopathic host bile [9]. Nucleotide sequences coding for kappa- (GAA51086) and zeta-type (GAA44819) GSTs have also been identified, but their protein identity and biological properties remain elusive.

GSTs are ubiquitously expressed in almost all known organisms [11]. Typical catalytic activity of GSTs is refined by the conjugation of glutathione (GSH; γ-Glu-Cys-Gly) to a wide variety of non-polar electrophilic, endogenous and exogenous toxic compounds [12]. GSTs play crucial roles against various toxicants, especially in helminth parasites that lack the cytochrome P-450 (CYP450) phase II detoxification enzyme. Most helminth GSTs can be classified into mu- and sigma-types [10, 13], although some GSTs demonstrate mosaic patterns of substrate/inhibitor specificity [14].

Omega-class GST (GSTo) is a relatively ancient cytosolic enzyme, but is the most recently characterized [11, 15]. A RNA polymerase-related protein designated stringent starvation protein A (SspA) represents a bacterial GST-like molecule due to its highly comparable structural property with GSTo, but lacks GST activity [16]. GSTo has interesting features compared with the other types of GSTs. GSTo has distinct enzymatic properties, e.g. GSH-dependent thioltransferase and dehydroascorbate reductase activity (DHAR), which might be attributable to its structural similarity to glutaredoxin [15]. GSTo shows high affinity toward *S*-hexylglutathione and 4-nitrophenyl acetate, but low affinity to 1-chloro-2,4-dinitrobenzne. GSTo utilizes cysteine residue to form a mixed disulfide bond with GSH, while most GSTs possess typical tyrosine or serine residues in the active site [17]. GSTo participates in modulation of calcium channels, interaction with cytokine inhibitory drugs, multistep biotransformation, signaling pathway during c-Jun N-terminal kinase (JNK)-mediated apoptosis, sequestration of hydrophobic substances/byproducts generated through diverse hepatic metabolisms and cellular protection from oxidative damages [17–19].

Helminth GSTos have been characterized from *Onchocerca volvulus* and *Schistosoma mansoni*, *Fasciola* spp. and free-living *Caenorhabditis elegans* [13, 20–22]. *Onchocerca volvulus* GSTo (*Ov*-GST3) identified by differential display RT-PCR demonstrates stress resistant effects [21]. Introduction of double-stranded RNA of the *Ov-*GST3 into mutant *C. elegans* induces resistance to oxidative stress [23]. Transgenic *C. elegans* that overexpress GSTo (GSTO-1) exhibits increased resistance during oxidative injuries [24].

In our previous study involving proteome analysis of *C. sinensis* GSTs, we observed that CsGSTos were inducible during stimulation of the worm with bile juice [9]. This result prompted us to further characterize biochemical features and biological functions relevant to the CsGSTos in response to oxidative stress. In this study, we characterized biochemical properties of two species of *C. sinensis* GSTos. We demonstrated that expression profiles of the CsGSTos were spatiotemporally regulated in accordance with the maturation of the worm’s reproductive system. We subsequently investigated possible biological protective roles of CsGSTos in these organs under oxidative stressful conditions.

## Methods

### Parasites

*Clonorchis sinensis* metacercariae were collected from naturally infected freshwater fish (*Pseudorasbora parva*) in an endemic area in Korea. Each of 100 metacercariae was orally infect to Sprague–Dawley rats. Worms were collected from the bile ducts at 1-, 2-, 3- and 4-weeks post-infection. Worms were washed more than 10 times with phosphate buffered saline (PBS, 100 mM, pH 7.4) at 4 °C and were stored at -80 °C. Fresh intact worms were immediately used in ex vivo stimulation experiments (see later section). Animals were housed in accordance with guidelines from the Association for the Assessment and Accreditation of Laboratory Animal Care.

### Cloning of *C. sinensis* omega-class GSTs

Expressed sequence tags (ESTs) were constructed through a screening of randomly selected clones of an adult *C. sinensis* cDNA library [25, 26]. The similarity patterns of the EST sequences were analyzed against the non-redundant database using BLASTX at the NCBI (http://www.ncbi.nlm.nih.gov). Clones showing high-level sequence identities with *Schistosoma mansoni* (AAO49385) and *Fasciola hepatica* (JX156880) GSTo were selected. The adult *C. sinensis* cDNA library was screened by PCR using vectors (T3 and T7 promoter primers) and gene-specific primers, which contained *Bam*HI (forward) and *Xho*I (reverse) restriction sites: CsGSTo1-forward (5′-CCG GAT CCA TGC CAA CCT GTT CCA AGC ATT TGC-3′); CsGSTo1-revese (5′-GGC TCG AGT TAC ATG TCC CAG TCA GGA TGA CCA-3′); CsGSTo2-forward (5′-ATG GAT CCA TGT GCT ATC TGG GAG ACG CAG GGA-3′); and CsGSTo2-reverse (5′-GGC TCG AGC TAG GCA ATT TCA AGA TTT GGC TTT CCA GC-3′). T7 promoter and forward primers were used to amplify the 3′-region; the T3 promoter and reverse primers were used for amplification of the 5′-region. The PCR thermal cycler profile included 35 cycles at 94 °C (50 s), 58 °C (50 s) and 72 °C (90 s), followed by 10 min extension at 72 °C. Amplicons were purified using a QIAquick PCR purification kit (Qiagen, Valencia, CA, USA), digested with respective enzymes and cloned into the pET-28a(+) vector (Novagen, Madison, WI, USA). The plasmids were transformed into *Escherichia coli* DH5α. Nucleotide sequences were determined from both strands. Two full-length cDNAs were obtained by overlapping the 5′- and 3′-region sequences.

### Bioinformatics

The coding profiles and the homology patterns were analyzed using the ORF Finder and BLAST programs. The functionally/structurally conserved domains were searched using ProfileScan (http://myhits.isb-sib.ch/cgi-bin/motif_scan). The secondary structure elements were predicted by the Jpred (www.compbio.dundee.ac.uk/jpred/). The Expasy-Sib Bioinformatics Resource Portal (http://web.expasy.org/compute_pi/) was used to predict the theoretical molecular mass (*M*_r_) and isoelectric point (p*I*). Tertiary structures were simulated using Swiss-PdbViewer (ver4.1) based on the human omega-class GST1 and 2 (pdb 1EEM and 3QAG). The amino acid sequences were employed as queries during sequence analyses using Hidden Markov Models (InterProScan, http://www.ebi.ac.uk/InterProScan/). The retrieved amino acid sequences were aligned with ClustalX 2.1 and optimized with GeneDoc (ver2.7) [27]. The phylogenetic tree was constructed using the neighbor-joining method and the molecular evolution genetics analysis (MEGA) ver5.1 software [28]. Statistical significance of each branching node was examined by a bootstrap analysis of 1000 replicates using SEQBOOT in the PHYLIP package [29].

### Expression of recombinant proteins

The full-length *CsGSTo1* and *2* cloned into the pET-28a(+) vector were introduced into *E. coli* BL21 (DE3). Expression of the recombinant proteins was induced with 0.1 mM isopropyl-β-D-thiogalactopyranoside (IPTG) for 4 h at 37 °C. Bacterial cells were sonicated and rCsGSTos were purified by Ni-nitrilotriacetic acid (NTA) affinity column (Qiagen) using Tris-HCl (50 mM, pH 8.0) supplemented with NaCl (200 mM) and imidazole (250 mM). His-tag was removed by a Thrombin CleanCleave kit (Sigma-Aldrich, St. Louis, MO, USA). The purified proteins were dialyzed against PBS (100 mM, pH 7.4) for 4 h at 4 °C, concentrated by lyophilization and analyzed by 12 % SDS-PAGE under reducing conditions.

### Specific antibodies and immunoblotting

Specific-pathogen free 6-week-old female BALB/*c* mice were immunized with rCsGSTos (100 μg each) emulsified with 2 % ammonium hydroxide gel adjuvant (InvivoGen, San Diego, CA, USA). Two weeks later, proteins (100 μg) mixed with emulsifier were boosted three times at one-week intervals. One-week later, the sera were collected and IgG fractions were purified using Protein G affinity column.

Proteins were separated by 12 % reducing SDS-PAGE and/or isoelectrically focused using IPG strips (pH 3–10; GE Healthcare, Piscataway, NJ, USA) for 30 kVh followed by 12 % SDS-PAGE (2-dimensional electrophoresis; 2-DE). The proteins transferred onto nitrocellulose membranes (Santa Cruz Biotechnology, Santa Cruz, CA, USA) were blocked in Tris-buffered saline (100 mM, pH 8.8) containing 0.01 % Tween 20 and 3 % skim milk for 1 h. The membrane was incubated with anti-rCsGSTo1 or 2 antibody (1:1,000 dilution) overnight at 4 °C and subsequently with horseradish peroxidase (HRP)-conjugated goat anti-mouse IgG (1:4,000 dilution; Cappel, West Chester, PA, USA) for 2 h. Signals were detected using West-Q Pico enhanced chemiluminescence (ECL) kit (GenDEPOT, Dallas, TX, USA). All images were obtained after 2 min exposure for quantitative analysis.

### Binding characteristics of native CsGSTos against *S*-hexylglutathione (SHG) and GSH

We determined the binding specificity of CsGSTos toward SHG and GSH. Adult *C. sinensis* (4-week-old) were homogenized with a Teflon-pestle homogenizer in PBS (100 mM, pH 7.4) containing a protease inhibitor cocktail (Roche, Basel, Switzerland). The supernatant was obtained by centrifugation at 20,000 g for 30 min at 4 °C. Proteins (200 μg protein per column) were loaded onto a SHG-agarose column (Sigma-Aldrich) or a glutathione-Sepharose 4B column (GE Healthcare). The columns were washed with 20 bed volumes of Tris-HCl buffer (50 mM, pH 7.8) containing 200 mM NaCl. Bound proteins were eluted using Tris-HCl buffer (50 mM, pH 7.8) with 0, 2 and 4 mM step-wise gradient fashions of SHG or GSH. Purified proteins resolved by 12 % SDS-PAGE/2-DE were stained with Coomassie brilliant G-250 (CBB) or further processed by immunoblotting using anti-rCsGSTo1 and 2 antibodies.

### Enzyme assay

GST activity was spectrophotometrically determined employing a panel of substrates (Sigma-Aldrich); 1-chloro-2,4-dinitrobenzene (CDNB; pH 6.5, 340 nm), 1,2-dichloro-4-nitrobenzene (DCNB; pH 7.5, 345 nm), 4-nitrobenzyl chloride (4-NBC; pH 6.5, 310 nm), 4-nitrophenyl acetate (4-NPA; pH 7.0, 400 nm), 4-hydroxy nonenal (pH 7.5, 340 nm), cumene hydroperoxide (CHP; pH 6.5, 340 nm) and ethacrynic acid (pH 7.5, 340 nm). The reactions were recorded for 5 min at 25 °C in 100 mM potassium phosphate buffer (pH 7.2, 200 μl) containing each 4 mM substrate and 4 mM GSH. The formation of ascorbate by the glutathione-dependent DHAR was detected in potassium phosphate buffer (50 mM, pH 7.2) supplemented with 1 mM GSH and 0.25 mM dehydroascorbate (DHA) at 265 nm. Thioltransferase activity was assayed using hydroxylethyl disulfide (HEDS, 2 mM) in potassium phosphate buffer (50 mM, pH 7.2) containing 0.2 mM NADPH, 0.5 mM GSH and 0.5 units of glutathione reductase for 2 min at 340 nm. One unit of enzyme activity was defined as the amount of enzyme that catalyzed the formation of one micromole of product per min in the presence of respective substrates. *V*max and app*K*m were determined by one site saturation assays in ranges of 0.01–5 mM DHA with 5 mM GSH (saturating concentration). We also used variable concentrations of GSH ranging from 0.01–5.0 mM with 5 mM DHA (saturating concentration). Enzyme activity was monitored by changes of absorbance and was converted to specific activity using a molar extinction coefficient (Δε = 5.3). Non-enzymatic reaction was concomitantly monitored and subtracted from the entire reaction rate. All enzyme assays were independently performed in triplicate at 25 °C. Data were analyzed by best fit algorithm in SigmaPlot10.0.1 (Systat, San Diego, CA, USA).

### Inhibition characteristics

We determined the inhibition mode of CsGSTos employing SHG and the anthelminthic drug praziquantel (PZQ; Shinpoong, Seoul, Korea). rCsGSTos (each 100 ng) were preincubated with Dulbecco’s PBS supplemented with 10–1,000 μM PZQ or 10–500 nM SHG for 2 min, after which the reaction was initiated by adding 1 mM GSH and 1 mM DHA. The increase in absorbance of the resulting GSH conjugate was recorded spectrophotometrically at 265 nm. A set of reactions under identical conditions was done for each inhibitor concentration and for controls. To examine the inhibition mode of the specific active sites, rCsGSTos were preincubated with saturating concentrations of GSH (5 mM) for 2 min before conjugating reaction with varying concentrations of DHA (0.01–5 mM). To determine the inhibition mode of inhibitors against the ligand-binding sites, the initial velocity of the enzyme reactions was observed in the presence of the respective inhibitors. rCsGSTos were incubated with saturating concentration of DHA (5 mM) for 2 min prior to the conjugating reaction with variable concentrations of GSH (0.01–5 mM). All measurements were independently done in triplicate. Data were analyzed by Lineweaver-Burk plots.

### Immunohistochemistry

In order to observe tissue distribution pattern of CsGSTos, immunohistochemical staining was done on adult worm sections. *Clonorchis sinensis* adult worms were fixed in 4 % neutral paraformaldehyde, embedded and cut into 4 μm-thick pieces. Sections were treated with 3 % H_2_O_2_ for 10 min, subsequently with Tris buffered saline (100 mM, pH 8.0) containing 3 % BSA and 0.1 % Tween 20 (TBS/T-BSA) for 1 h. The slides were incubated overnight at 4 °C with anti-rCsGSTo1 or 2 antibody (1:400 dilution in TBS/T-BSA) and further incubated with HRP-conjugated goat anti-mouse IgG (1:1,000 dilution; Cappel). Color reactions were developed using HighDef blue chromogen (Enzo Life Sciences, Farmingdale, NY, USA) with PBS (100 mM, pH 7.4) supplemented with 0.05 % 3,3'-diaminobenzidine blue and 0.015 % H_2_O_2_ for 5 min. The images were photographed under a TissueFAXS plus (TissueGnostics, Vienna, Austria).

### In vitro induction of CsGSTos under oxidative stresses

To assess biological reactivity of CsGSTos under oxidative stressful conditions, we observed induction profile of CsGSTos upon treatment with oxidizing chemicals. Fresh intact worms were stabilized for 1 h at 37 °C in 5 % CO_2_ atmosphere in serum- and phenol red-free RPMI medium. Worms (10 worms per group per 1 ml of medium) were transferred into fresh medium containing different doses of 5-hydroxy-1,4-naphthoquinone (Juglone; Sigma-Aldrich) (25–100 μM) or CHP (1–4 mM) and incubated for 1 h at 37 °C. The worms were harvested and fractionated into individual compartments, such as seminal receptacle, vitelline follicle-enriched parenchyma and eggs under a dissecting microscope. The conditioned medium containing excretory-secretory products (ESP) was also harvested and sperm was separately collected under a dissecting microscope. Proteins of the respective compartments were extracted in PBS (100 mM, pH 7.4) containing protease inhibitor cocktail (one tablet/25 ml PBS; Complete; Roche) and centrifugation at 12,000 *g* for 30 min at 4 °C. The proteins prepared from individual compartments of the worms incubated without oxidative treatment were used as controls. Cs tubulin (CsTub; DF143021), whose constant expression was verified by RT-PCR in association with the respective stimuli [26], was used as an internal control. The induction profiles of CsGSTo were examined by immunoblotting probed each with anti-rCsGSTo antibody and by quantitative real-time RT-PCR (qRT-PCR). At least three independent experiments were done with freshly prepared worms.

### Reverse-transcription PCR (RT-PCR) and qRT-PCR

Expressional changes of CsGSTos during worm’s maturation and in response to oxidative stress were determined. Total RNA was extracted from the experimental worms or different developmental stages of worms using a RNeasy Mini kit (Qiagen). RT-PCR was done using RT-PCR PreMix kit (iNtRON, Seongnam, Korea). *CsGSTo* transcripts in the RNA (1 μg) were amplified by PCR with the following primers: CsGSTo1-forward, 5′-GTT TCC ATT TGT GGA C-3′ and -reverse, 5′-TGG TAG CTG CAA TAC G-3′; CsGSTo2-forward, 5′-TCG TTT GAG CGA ATC G-3′ and -reverse, 5′-CAG CGA GAC TGA GTT G-3′. The thermal cycler profile included pre-heating for 30 min (50 °C) and 10 min (94 °C), 30 cycles of 50 s (94 °C), 50 s (58 °C) and 90 s (72 °C) with 10 min final extension (72 °C). *CsTub* gene was amplified by PCR using gene specific forward primer (5′-ATT CAG CTG TCC TGG GAA AC-3′) and reverse primer (5′-ACT GCA TTG ATA ACG AAG CG-3′). Thermal cycler profile included 25, 30 and 35 cycles at 94 °C (50 s), 58 °C (50 s) and 72 °C (90 s), followed by a 10 min final extension (72 °C). The PCR products were analyzed on 1 % agarose gel with ethidium bromide staining.

qRT-PCR was conducted using the Rotor-Gene SYBR Green PCR kit and the Rotor-Gene Q Real-time PCR (Qiagen). Total RNA (200 ng) treated with DNase was reverse-transcribed into cDNA using the SuperScript First-Strand Synthesis system (Thermo Fisher Scientific, Waltham, MA, USA). The cDNA was used to examine transcriptional activities of *CsGSTo* genes with specific primers. The qRT-PCR program included pre-denaturation for 5 min (94 °C), 40 cycles of amplification (94 °C for 15 s, 60 °C for 30 s and 72 °C for 30 s) and a melt cycle from 65 °C to 95 °C. Control reactions were done with RNAs that had not been reverse-transcribed. mRNA abundancy was evaluated in three independent samples for each group with three technical repeats. Data were normalized against those obtained with the *CsTrop* (ΔC_T_). Fold inductions (ΔΔC_T_) were calculated by comparison of the non-stimulated controls. Data were analyzed with the Rotor-Gene Q ScreenClust HRM software using the 2^-ΔΔC^_T_ method [30].

### Disc diffusion, cell growth and survival assays under oxidative stress

We observed effects of CsGSTo during oxidative stress employing CsGSTo overexpressing *E. coli. Escherichia coli* BL21 cells transformed with *CsGSTo1* or *2* expression plasmids and control cells transformed with mock vector were induced for expression of recombinant proteins by adding 0.1 mM IPTG for 4 h at 37 °C. The cells (5 × 10^8^) were cultured on LB-kanamycin agar plates for 1 h at 37 °C. Discs (6-mm diameter) soaked with 10, 50, 100 and 200 mM CHP or Juglone were placed on the surface of the top agar. The cells were grown for 24 h at 37 °C and the inhibition zones were measured. For the cell growth assay, stationary-phase cultures of CsGSTo overexpressing bacterial cells and control cells were diluted to an optical density at 600 nm (OD_600_) of 0.004. The cells were grown in LB broth at 37 °C until exponential phase (OD_600_ = 0.13–0.14). Aliquots were treated with 0.5, 1, 2 and 4 mM CHP. Growth curves were obtained in Erlenmeyer flasks at 37 °C and 225 rpm. The cultures were diluted to an OD_600_ of 0.01 in LB broth and the OD_600_ was measured every 1 h for 25 h. CsGSTo overexpressing *E. coli* and control cells were subjected to survival assay against oxidative injury. *Escherichia coli* cells were grown (OD_600_ = 0.5), after which CHP or Juglone was added to exponential bacterial suspension (0.49 ml) to final concentrations of 1, 2 and 4 mM, and incubated from 20–60 min at 37 °C with shaking (225 rpm). The cells diluted in PBS (0.1 ml) were plated on LB agar plates and grown for 48 h at 37 °C. Cell viability was determined by counting colony-forming units per ml (CFU/ml) as percentage of surviving cells compared to untreated cells. The limit of detection was 100 CFU/ml. All assays were done independently in triplicate.

### Statistical analyses

Data are expressed as mean ± standard deviation (SD) of 3–5 independent experiments. Statistical significance was evaluated by a one-way analysis of variance (ANOVA) using the Statistical Package for the Social Sciences (SPSS; ver20.0) software (SPSS, Chicago, IL, USA), or Student’s *t-*test followed by a Bonferroni correction, as appropriate. Differences in mean values were considered statistically significant at *P* < 0.05.

## Results

### Molecular properties of *C. sinensis* omega-class GSTs

**Fig. 1:**
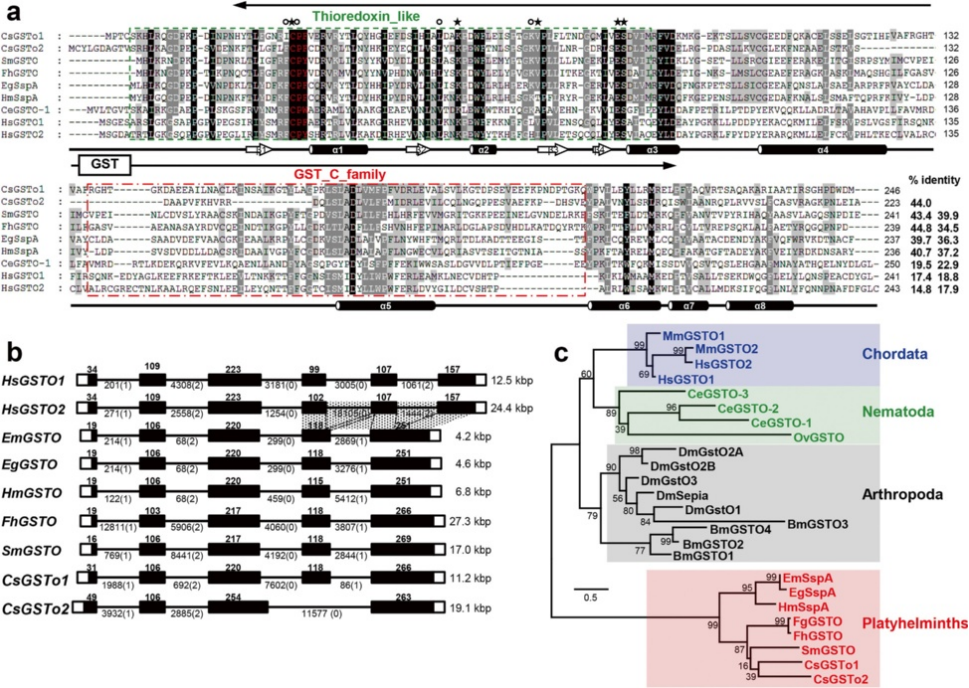
Structural property and phylogenetic position of *C. sinensis* omega GST1 and 2 (CsGSTo). **a** Comparison of primary structure of CsGSTo1 and 2 with other related members. Residues directly contacting glutathione are indicated by asterisks. Glutathione-binding residues are marked by closed circles. Cysteine residues that constitute the active site of omega GSTs are denoted by red letters. Putative thioredoxin and GST_C domains are indicated by dotted green- and red-boxes. Dots represent gaps introduced into the sequences to optimize sequence identities. CsGSTo1, *Clonorchis sinensis* omega GST1 (KX163088); CsGSTo2, *C. sinensis* omega GST2 (KX163089); SmGSTO, *Schistosoma mansoni* omega GST (AAO49385); FhGSTO, *Fasciola hepatica* omega GST (JX156880); EgSspA, *Echinococcus granulosus* stringent starvation protein A (CDJ25309); HmSspA: *Hymenolepis microstoma* stringent starvation protein A (CDJ10775); CeGSTO-1, *Caenorhabditis elegans* omega GST (GAA34234); HsGSTO1 and 2, *Homo sapiens* omega GST1 and 2 (AAF73376 and AAH56918). Gene names are adapted from the GenBank database (http://www.ncbi.nlm.nih.gov/). **b** Comparison of genomic structure of *CsGSTo1* and *2* with platyhelminth and human orthologues. Coding DNA sequences are presented with solid squares in proportion to their relative sizes. The 5′- and 3′-untranslated regions are marked with open squares (voluntary length). Intervening introns are shown by solid lines (fixed length). Numerals in parentheses indicate the phase of each intron. The lengths of exons and introns in bp are presented. The dotted boxes show exons, which have acquired introns during evolution of paralogous/orthologous genes. **c** Phylogenetic relationships of CsGSTos. The phylogenetic position of CsGSTos was predicted on the basis of alignment of amino acid sequences. The tree was constructed by the neighbor-joining algorithm of PHYLIP. Numbers at the major branching nodes demonstrate their percentages of appearance in 1,000 bootstrap replicates. GenBank accession numbers of missing entities in **a** include FgGSTO, *Fasciola gigantica* omega GST (AFX98105); EmSspA, *Echinococcus multilocularis* stringent starvation protein A (EmuJ_000919600); CeGSTO-2, *Caenorhabditis elegans* omega GST-2 (CCD62560); CeGSTO-3, *C. elegans* omega GST-3 (CCD72880); OvGSTO, *Onchocerca volvulus* omega GST (AAF99575); BmGSTO1, *Bombyx mory* omega GST1 (NP_001040131); BmGSTO2, *B. mory* omega GST2 (NP_001037406); BmGSTO3, *B. mory* omega GST3 (NP_001040435); BmGSTO4, *B. mory* omega GST4 (NP_001108461); DmGstO1, *Drosophila melanogaster* omega GST-1 (NP_648237); DmSepia, *D. melanogaster* Sepia (NP_648235); DmGstO2A, *D. melanogaster* omega GST2A (NP_729388); DmGstO2B, *D. melanogaster* omega GST2B (NP_648236); DmGstO3, *D. melanogaster* omega GST3 (NP_648235)

We isolated two full-length cDNAs (1,965 bp and 1,061 bp) that putatively coded for *C. sinensis* GSTs through amplification of 5'- and 3'-regions using the cDNA library. The deduced proteins were composed of 246 and 223 amino acids, respectively, with predicted *M*_r_*/*p*I* of 28,183 Da/5.83 and 26,344 Da/5.64, respectively. They harbored characteristic features of cytosolic GST superfamily, such as N-terminal thioredoxin-like domain (βαβαββα) and C-terminal α-helical domains. The N-terminal thioredoxin-like domain appeared to be more tightly conserved (25.6–57.8 %) among related members compared to the C-terminal GST-C domains (14.6–39.2 %) (Fig. 1a). We designated these cDNAs as *C. sinensis* omega-class GST1 (CsGSTo1) and 2 (CsGSTo2) and registered them in GenBank under accession numbers KX163088 and KX163089.

When we simulated the tertiary structure of these proteins, a cysteine residue that constituted the omega-class specific active site (C30/C36) and glutathione binding amino acids were recognized in appropriate positions (K57/K63, V70/V76, E83/E88 and S84/S89 for CsGSTo1 and 2, respectively). Despite their low sequence identity (14.8–18.8 %), the general topology of CsGSTos was comparable with human GSTos. However, additional amino acids between α4 and α5 helices (CsGSTo1) and the N-terminal extension (CsGSTo2) were observed. These appeared to form an unstructured loop-like domain, which could not be readily determined (Additional file 1: Figure S1a, b).

The genomic structure of *CsGSTo1* spanned 11.2 kbp with five exons and four intervening introns. The 19.1 kbp-coding DNA sequence of *CsGSTo2* was split into four exons by three introns. In contrast to *C. sinensis*, only a single gene orthologous to CsGSTo was retrieved from another platyhelminth examined. Comparison of *CsGSTo* structures with other trematode orthologues revealed that a single exon corresponding to the fourth exon of *CsGSTo1* was deleted in *CsGSTo2*. The first three introns of the trematode genes were also detected in the orthologous positions of human genes. However, the fourth intron (and fifth intron of human genes) appeared to be introduced after the divergence of Chordata (Fig. 1b). A phylogenetic tree suggested that the *GSTo* gene has undergone donor organism-specific duplication event(s), at least in *C. sinensis* and some kinds of ecdysozoan invertebrates (Fig. 1c).

### Identification of the native CsGSTos by affinity binding to SHG and GSH

**Fig. 2:**
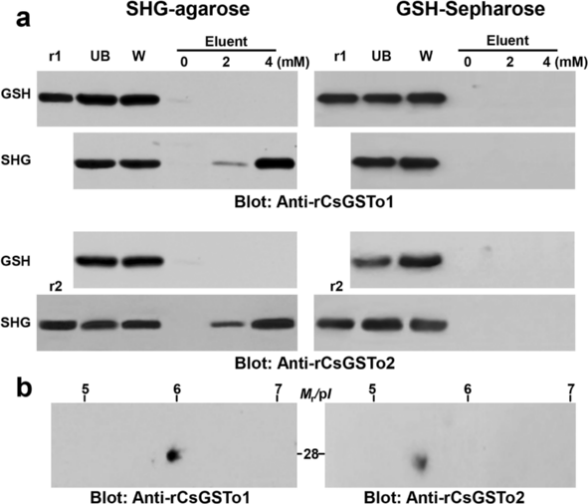
Binding affinity of the native CsGSTos toward *S*-hexylglutathione (SHG) and reduced glutathione (GSH). **a** *C. sinensis* adult extracts (200 μg) bound each with SHG-bead and glutathione-Sepharose 4B were eluted using 4 mM SGH or 4 mM GSH. The bound proteins (100 ng) were separated by 12 % reducing SDS-PAGE, transferred to nitrocellulose membranes and probed with anti-rCsGSTo1 and 2. The membranes were developed with ECL. r1 and r2, rCsGSTo1 and 2 (each 100 ng) loaded as positive controls. UB, unbound fractions of *C. sinensis* extracts; W, washing fractions; Eluent, bound fractions eluted with 0, 2 and 4 mM SHG or GSH. **b** 2-DE profile of native CsGSTo1 and 2. The bound proteins of SHG-agarose bead (10 μg) were isoelectrically focused using IPG strip (pH 3–10), after which resolved by 12 % SDS-PAGE and blotted onto nitrocellulose membrane. The membranes were incubated with anti-rCsGSTo1 and 2 antibodies (1:1,000 dilution) and subsequently with HRP-conjugated goat anti- mouse IgG (1:4,000 dilution). The blots were developed with ECL

Binding affinity of native CsGSTos to SHG and GSH was assessed. When *C. sinensis* adult extracts bound to SHG-agarose matrix were eluted with 4 mM SHG, considerable amounts of bound proteins were eluted, while those eluted with GSH did not contain bound proteins. Conversely, when glutathione-Sepharose 4B beads were used as a binding partner, no protein was eluted with either GSH or SHG (Fig. 2a). This result suggested strongly that CsGSTos have very low affinity to GSH compared to SHG, which has more hydrophobic alkyl groups [20, 31], and agreed with the app*K*m values. The app*K*m value of SHG was remarkably higher (3 × 10^5^-fold) than that of GSH (see below). We also separated bound proteins by 2-DE, transferred to nitrocellulose membrane and probed with respective antibodies. Reactive signals were observed approximately at 28 kDa with p*I* 5.9 (CsGSTo1) and 27 kDa with p*I* 5.6 (CsGSTo2) (Fig. 2b), which matched well with those predicted from the primary sequences. When we analyzed 2-DE/immunoblotting profile of adult extracts, a similar result was observed (data not shown).

### Enzymatic properties of CsGSTos

rCsGSTos purified by Ni-NTA chromatography followed by thrombin cleavage migrated to approximately 28 and 27 kDa and showed an antibody response specific to the respective antibodies (Additional file 2: Figure S2a, b). We used these proteins during characterization of enzyme property. The enzymatic reactions catalyzed by rCsGSTos followed Michaelis-Menten kinetics when one of the co-substrates was provided at a saturating concentration. The enzymes showed a relatively high activity toward the omega-class specific substrate, 4-NPA (*V*max = 0.84 ± 0.06 and 0.73 ± 0.04 μmol/min/mg), but showed a low affinity to CDNB. Interestingly, rCsGSTo1 and 2 exhibited high enzyme activity against mu- and theta-specific substrate, 4-NBC (*V*max = 1.42 ± 0.09 and 0.91 ± 0.24 μmol/min/mg, respectively). Conversely, rCsGSTo1 and 2 revealed no enzyme activity toward other-types of GST substrates, such as CHP (alpha), ethacrynic acid (pi), DCNB (mu) and 4-hydroxy nonenol (alpha and theta) (Table 1).

**Table 1:** Substrate specificity of recombinant CsGSTo1 and 2

	Substrate	Class-specificity	Specific activity (μmol/min/mg)	
			rGSTo1	rGSTo2
GST-specific	CDNB^a^	All	0.13 ± 0.01	0.10 ± 0.02
	Cumene hydroperoxide	α	nd^b^	nd
	DCNB^c^	μ	nd	nd
	Ethacrynic acid	π	nd	nd
	4-nitrobenzyl chloride	μ and θ	1.42 ± 0.09	0.91 ± 0.24
	4-nitrophenyl acetate	ω	0.84 ± 0.06	0.73 ± 0.04
	4-hydroxy nonenal	α and θ	nd	nd
DHAR^d^	Dehydroascorbate	ω	1.16 ± 0.04	1.08 ± 0.02
Thioltransferase	Hydroxyethyl disulfide	ω	1.08 ± 0.02	0.76 ± 0.02

^a^1-chloro-2,4-dinitrobenzene

^b^Not detected

^c^1,2-dichloro-4-nitrobenzene

^d^Dehydroascorbate reductase

DHAR and thioltransferase activities of rGSTos were determined. rGSTo proteins demonstrated considerable reactivity against DHA and HEDS (Table 2). The *V*max values for rCsGSTo1 and 2 against DHA were 1.16 ± 0.02 and 1.08 ± 0.02 μmol/min/mg, and app*K*m values were 0.21 ± 0.02 and 0.17 ± 0.02 mM, respectively. The *V*max values for GSH catalyzed by rCsGSTo1 and 2 were estimated to be 0.42 ± 0.04 and 0.56 ± 0.08 μmol/min/mg, with app*K*m values of 0.19 ± 0.02 and 0.16 ± 0.02 mM, respectively. These enzymes showed a maximal DHAR activity at 25 °C with optimal pH of 7.6 (rCsGSTo1) and 7.2 (rCsGSTo2) (Additional file 3: Figure S3a, b).

**Table 2:** Kinetic parameters for recombinant CsGSTo1 and 2

Kinetic parameter		rCsGSTo1	rCsGSTo2
DHA	*V*max	1.16 ± 0.02 μmol/min/mg	1.08 ± 0.02 μmol/min/mg
	*K*m	0.21 ± 0.02 mM	0.17 ± 0.02 mM
	*K*cat	0.48 s	0.43 s
	*K*cat/*K*m	2.29 × 10^3^/s/M	2.53 × 10^3^/s/M
GSH	*V*max	0.42 ± 0.04 μmol/min/mg	0.56 ± 0.08 μmol/min/mg
	*K*m	0.19 ± 0.02 mM	0.16 ± 0.02 mM
	*K*cat	0.37 s	0.33 s
	*K*cat/*K*m	1.95 × 10^3^/s/M	2.06 × 10^3^/s/M

### Inhibition characteristics of SHG and PZQ against hydrophobic ligand- and glutathione-binding sites

**Fig. 3:**
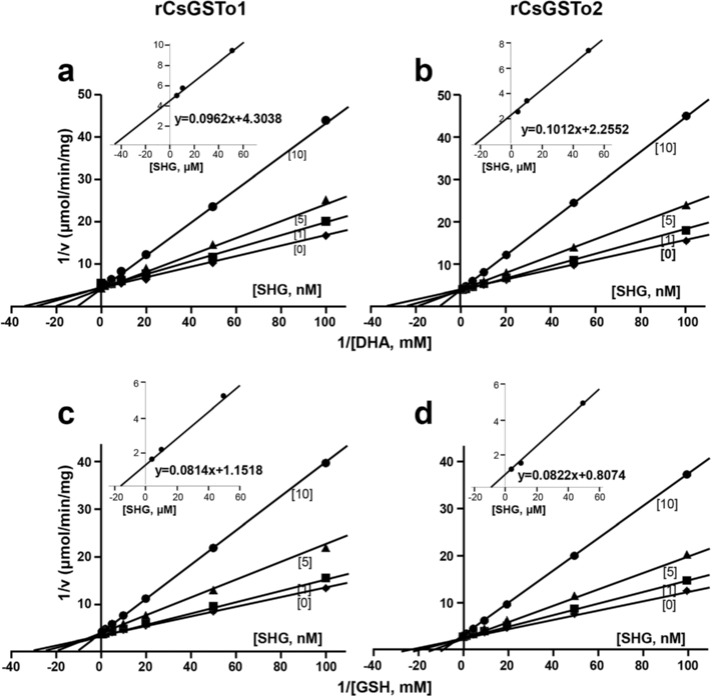
Steady-state kinetics of *S*-hexylglutathione (SHG) against rCsGSTo1 and 2. Lineweaver-Burk plot of inhibition mode of initial velocities of rCsGSTo1 and 2. **a**, **b** Activities (1/v) *versus* 1/[DHA] (mM^-1^) or rCsGSTo1 and 2. **c, d** Activities (1/v) *versus* 1/[GSH] (mM^-1^) in the absence (*diamond*) and presence of 1 nM (*rectangle*), 5 nM (*triangle*) and 10 nM (*circle*) of SHG. Variable concentrations of DHA and GSH from 0.01 to 100 mM were applied. Data are plotted in double reciprocal form. Insets show secondary plot of the 1/*V*max values obtained from the primary Lineweaver-Burk plot *versus* SHG concentration for the determination of *K*i value. All assays were independently done in triplicate (*n* = 3, mean ± standard deviation, SD) and representative figures are shown

SHG potently and competitively inhibited both the DHA and GSH during binding of the H- and G-sites (Fig. 3). SHG might act via nucleophilic attack of the active site cysteine on the cysteinyl sulfur of the SHG to form a mixed disulfide with the DHA/GSH moiety with high affinity [32]. In contrast, PZQ, an anthelminthic drug, displayed non-competitive inhibition of both DHA and GSH (Additional file 4: Figure S4). PZQ might bind to a site other than the active- and glutathione-binding sites and act allosterically. We determined IC_50_ values of rCsGSTos against SHG and PZQ at a saturating concentration (IC_50_SC). SHG demonstrated IC_50_SC values at nanomolar concentration; 0.84 ± 0.06 and 0.79 ± 0.04 nM for rCsGSTo1 and 2, respectively, while PZQ revealed high values at micromolar concentrations (Additional file 5: Table S1).

### Spatiotemporal expression patterns of CsGSTos

**Fig. 4:**
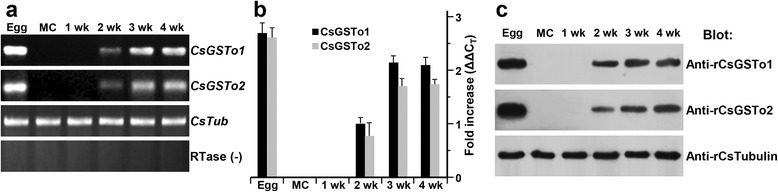
Expression profiles of the *C. sinensis* GSTo1 and CsGSTo2 according to developmental stages. **a** Total RNAs (1 μg) extracted from each of the developmental stages were reverse transcribed as indicated on top. The mRNA transcripts of CsGSTo1 and 2 amplified by a semiquantitative RT-PCR were analyzed by 2 % agarose gels with ethidium bromide staining. *C. sinensis* tubulin gene (*CsTub*), which was shown to be constitutively expressed throughout developmental stages, was used as a control. The reaction mixture that did not contain reverse transcriptase during synthesis of the first single strand cDNA was used as a negative control. *Abbreviations*: Egg, *C. sinensis* egg; MC, metacercaria; 1 wk, 1-week-old juvenile; 2 wk, 2-week-old juvenile; 3 wk, 3-week-old immature; 4 wk, 4-week-old-mature *C. sinensis*. **b** Alteration of *CsGSTo* transcripts by qRT-PCR. The mRNA transcripts in each of the RNA samples (200 ng) were reverse-transcribed and the resulting cDNAs were employed in qRT-PCR as templates. The fold increase was calculated by differences in threshold cycles (ΔΔC_T_) of the CsGSTo1 and 2 among different developmental stages. *CsTub* gene was used as normalization control. **c** Expressional changes of CsGSTo1 and 2 proteins determined by immunoblotting probed with anti-rCsGSTo1 and 2. *C. sinensis* tubulin (CsTub) was employed as a control. Each lane contained 100 ng protein

We observed expression patterns of CsGSTos following worm maturation. The transcription of *CsGSTo1* and *2* mRNAs was initiated in 2-week-old juveniles and increased rapidly through 3-week-old immature and 4-week-old-mature *C. sinensis*, but these genes were not expressed in the metacercaria and the 1-week-old juvenile stage. The expression of CsGSTos was highly upregulated in eggs (Fig. 4a, b). The upregulated expression profiles of CsGSTo proteins according to maturation of *C. sinensis* were similarly observed as those of transcripts (Fig. 4c).

**Fig. 5:**
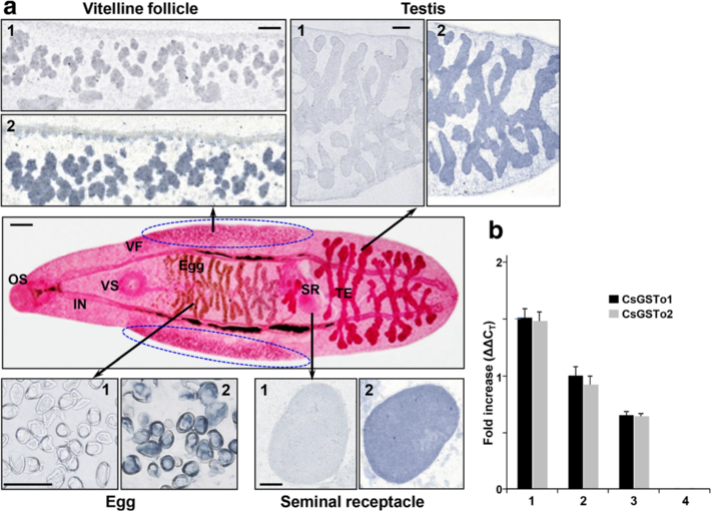
Immunolocalization of *C. sinensis* GSTo1 and compartmental expression of *C. sinensis GSTo1* and *2* transcripts. **a** Adult worm sections (thickness 4-μm) were incubated with preimmune mouse serum (panels 1) or anti-rCsGST1 antibody (panels 2) at a same dilution ratio (1:200) and subsequently incubated with HRP-conjugated goat anti-mouse IgG antibody (1:1,000 dilutions). The slides were developed with the blue-immunohistochemistry chromogen 3,3'-diaminobenzidine blue supplemented with H_2_O_2_. Acetocarmine-stained in toto specimen is also seen. The regions marked by dotted-ellipses were separately prepared as vitelline follicle-enriched parenchyma for Western blot (see also Fig. 7). *Abbreviations*: OS, oral sucker; IN, intestine; VS, ventral sucker; VF, vitelline follicles; SR, seminal receptacle; TE, testis. *Scale-bars*: 50 μm. **b** Expression of *CsGSTo* transcripts in *C. sinensis* reproductive system by qRT-PCR. Total RNAs (each 200 ng) extracted from respective organs were reverse transcribed into cDNA and subjected to qRT-PCR. The fold increase was calculated by differences in threshold cycles (ΔΔC_T_). *Key*: 1, egg; 2, whole worm; 3, testis, seminal receptacle and vitelline follicle-enriched fractions; 4, parenchymal fractions without reproductive organs

We determined the distribution patterns of CsGSTo proteins. CsGSTo1 protein demonstrated histological locality to the eggs, vitelline follicles, seminal receptacle and testes (Fig. 5a). Other organs/tissues showed no positive reaction. When we examined *CsGSTo* transcripts in different organs/tissues of the worm, expression of *CsGSTo* mRNAs was evident in eggs and reproductive organs, such as vitelline follicles, seminal receptacle and testes. Conversely, transcription of these genes was not observed in parenchymal tissue excluding eggs and reproductive organs (Fig. 5b). Immunostaining with anti-rCsGSTo2 antibody revealed positive reactions similar to those with anti-rCsGSTo1 antibody (data not shown). These results collectively indicate that CsGSTos are abundantly expressed in the reproductive system with an upregulated fashion according to the sexual maturation of the worms in the definitive host.

### Induction profile of CsGSTo transcripts and proteins under oxidative stress

**Fig. 6:**
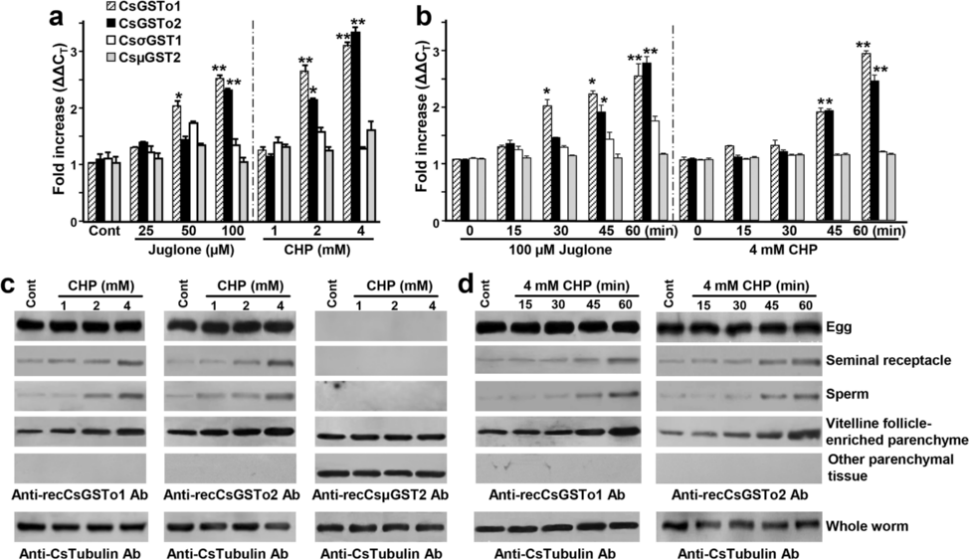
Induction profiles of *C. sinensis* GSTo transcripts and proteins under oxidative stress conditions. **a** The fresh live adults (10 worms per group) were stabilized in serum-free RPMI media for 1 h at 37 °C and were treated with Juglone (25–100 μM) or cumene hydroperoxide (CHP; 1–4 mM) for 1 h at 37 °C. Total RNAs (200 ng) were reverse-transcribed and subjected to qRT-PCR. The fold increase was calculated by differences in threshold cycles (ΔΔC_T_). **b** The worms were incubated in the presence of Juglone (100 μM) or CHP (4 mM) from 15–60 min as indicated. Total RNAs (200 ng) were extracted and fold increase (ΔΔC_T_) of each transcript was examined by qRT-PCR. *P < 0.05; **P < 0.01. **c**, **d** Proteins (20 μg) extracted from respective compartments the parasites incubated with different doses of CHP (1–4 mM) and different time intervals (15–60 min) were separated by 12 % reducing SDS-PAGE, blotted to nitrocellulose membranes and probed with anti-rCsGSTo1 and 2, respectively. *C. sinensis* tubulin, which did not show expressional changes upon oxidative stimuli, was used as a control. Positive signals were detected with ECL. Vitelline follicle-enriched fractions were prepared from the bilateral margins of middle portions of the worm, as marked in Fig. 5

Adult *C. sinensis* were incubated with different doses of Juglone or CHP for 1 h. Expression of *CsGSTo1* and *2* was significantly augmented (Fig. 6a). When we determined temporal induction of these genes, activation profiles of the *CsGSTo* genes were somewhat differentially regulated in response to Juglone or CHP. CsGSTo1 showed a relatively sensitive response to Juglone at an early time point with low dosages compared to *CsGSTo2*, but CHP acted on both genes at later times. Induction of CsGSTos was detected after 45 min of incubation, when treated with 4 mM CHP (Fig. 6b). The *CsμGST2* gene used as the negative control [9] did not show altered transcription levels.

Worms treated with different dosages and for different times were harvested and fractionated into individual compartments including eggs, vitelline follicle-enriched parenchyma (see also Fig. 5a), seminal receptacle and sperm. When Western blots were probed with anti-rCsGSTo1 and 2 antibodies, expression of CsGSTo1 and 2 proteins appeared to gradually increase in a dose-dependent manner (Fig. 6c). Expression of CsGSTo proteins was also upregulated with time-lapse, although the increasing levels could not be precisely determined (Fig. 6d). Interestingly, expression of GSTos in eggs was maintained at high levels regardless of oxidative conditions and was not changed by the oxidizing chemicals. Expressional changes of CsμGST2 (negative control) were not observed as previously reported [9]. Cs tubulin, employed as an internal control, also did not show expressional changes (Fig. 6c, d).

### CsGSTo overexpressing *E. coli* are resistant to oxidant-mediated killing

**Fig. 7:**
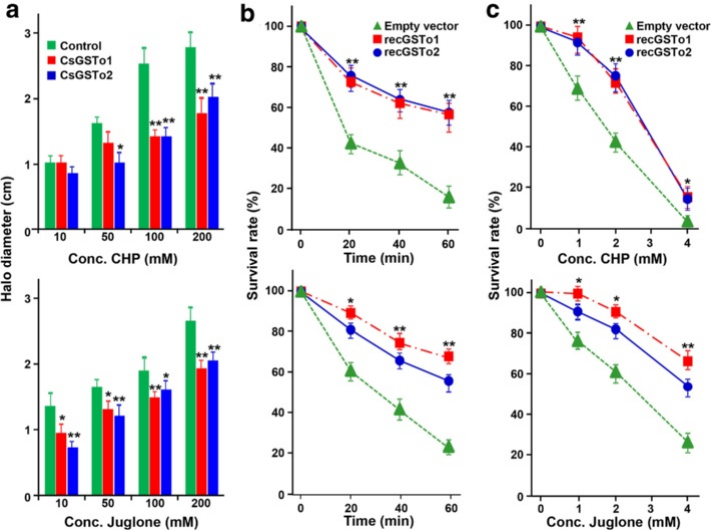
Protective roles of CsGSTos in *E. coli* transfected with CsGSTo expression plasmids under oxidative stress. **a** Disc diffusion assays using CsGFSTos overexpressing *E. coli*. LB agar was overlaid with top agar containing 5 × 10^8^ *E. coli* cells transfected with recombinant CsGSTo plasmids or mock vector. Filter-discs soaked with 10, 50, 100 and 200 mM of cumene hydroperoxide (CHP) or Juglone were placed on the plate and incubated overnight, after which the inhibition zones (halo diameter) were measured. *P < 0.05; **P < 0.01. **b** Effect of CHP (upper panel) or Juglone (lower panel) on the survival of exponentially growing *E. coli*. Cells were incubated for 60 min at 37 °C in the presence of 2 mM CHP or Juglone. **c** Survival curves of *E. coli* following exposure to different doses of CHP or Juglone (1, 2 and 4 mM) for 20 min at 37 °C. *P < 0.05; **P < 0.01. Data shown represent mean ± standard deviation, SD (*n* = 3)

CsGSTo overexpressing *E. coli* cells were grown overnight in the presence of filter discs soaked with different doses of CHP or Juglone, after which halo diameters were measured. The killing zones were significantly smaller than those of control cells (29–36 % halo reduction; *F*_(3, 16)_ = 35.9, *P* < 0.01) (Fig. 7a and Additional file 6: Figure S5a). CsGSTo overexpressing *E. coli* showed 1.2- to 2-fold increase in growth compared to control cells when exposed to 0.5, 1 and 2 mM CHP. However, cell growth of CsGSTo overexpressing *E. coli* and control cells were significantly suppressed in the presence of high concentrations (4 mM) of CHP. Similar results of halo reduction and cell growth patterns were evident upon treatment with Juglone, although its effects seemed to be less prominent compared to those of CHP (Additional file 6: Figure S5b). We next investigated cell survival rate of CsGSTo overexpressing *E. coli* cells. When treated with 2 mM CHP, cell survival was 52.6–55.8 %, compared to the 15.2 % survival of control cells after 1 h exposure (*F*_(3,16)_ = 991.0, *P* < 0.01) (upper panel, Fig. 7b). Similar results were observed during incubation with different doses of Juglone (lower panel, Fig. 7b). The survival rates of both cells were time- and dose-dependently decreased, but CsGSTo overexpressing cells demonstrated 1.4- to 5.5-time greater resistance compared to control cells (Figs. 7b, c).

## Discussion

In this study, we characterized two GSTos of the liver fluke *C. sinensis*, which showed differential and common biochemical and biological properties compared to GSTos characterized from other organisms including helminths. Analysis of expression patterns of CsGSTos in different developmental stages and in the different tissues, as well as induction profile under oxidative stress might provide information regarding functional relevance of CsGSTos. Uniquely, *C. sinensis* possessed two paralogous GSTos, which exhibited high activity toward 4-NBC, a mu- and theta-specific substrate. Expression of CsGSTos was spatiotemporally regulated along with maturation of the worm’s reproductive system. They showed augmented expression in response to oxidative stress and suggested their biological impacts in those organs. CsGSTos shared common properties with other homologous enzymes, such as harboring a cysteine residue in the active sites to form mixed disulfide bond [7], DHAR/thioltransferase activities and a weak conjugation activity of CDNB. They also have high binding affinity to SHG [15, 20]. Finally CsGSTos are involved in the cellular protection from oxidative injuries [22, 24].

Interestingly, rCsGSTos demonstrated a significant enzyme activity toward the mu- and theta-class specific substrate, 4-NBC (Table 1). GSTos characterized from humans (HsGSTO1), *Anopheles cracens* (AcGSTO1-1) and *S. mansoni* (SmGSTO) do not show conjugating activity toward 4-NBC [20, 33, 34]. A recent genome-wide survey of *C. sinensis* revealed that the parasite harbors 12 GST species, which are segregated to omega-, zeta-, mu-, sigma-, kappa- and membrane-associated protein in eicosanoid and glutathione metabolism-like protein (MAPEG), while theta-class GST(s) is not recognized [10, 20, 35]. Previous studies involving proteome analyses of *F. hepatica* and *F. gigantica* GSTs also did not identify theta-class GST [13, 36]. During a survey of *S. mansoni*, *F. hepatica* and *F. gigantica* genomes in the wormbase (http://parasite.wormbase.org/), we could not retrieve theta-class GST(s). Since GSTos are thought to be intermediate forms, which have occurred between the ancient glutaredoxin and the later mu- and/or theta-class GSTs [34], it is possible that CsGSTos might retain additional roles substitute for theta-class GST. Alternatively, CsGSTos might acquire a unique catabolic activity toward 4-NBC to ensure its physiological demand during maturation of the reproductive system. To address this intriguing phenomenon, identification of theta-class GST(s) and 4-NBC catalyzing activity of mu-class GSTs of diverse platyhelminths deserve further studies.

The differential expression of organ-specific transcripts of GSTos in higher mammals and insects has been well described [17, 37–40]. The expression of helminth GSTos also appeared to be determined by expression of tissue- and/or stage-specific transcripts of different helminths including a free-living nematode. *Onchocerca volvulus* GSTo was expressed only in the egg shell at the morula stage of the developing embryo [22]. *Caenorhabditis elegans* GSTo was expressed in the intestinal cells of the late embryo and adult hermaphrodite, where intestine specific GATA-type transcription factor (*Elt-2*) was predominantly expressed [24]. The expression of CsGSTos was detected to begin in 2-week-old juveniles and rapidly increased up to the 4-week-old adult stage and the highest expression was observed in eggs. CsGSTos were abundantly distributed in the reproductive system, such as vitelline follicles, testes, seminal receptacle and eggs (Fig. 5). This result demonstrated that expression regulation of CsGSTos is associated with the development of the reproductive system. The genuine sexual maturation of *C. sinensis* reportedly begins in juveniles between 1- and 2-weeks [26]. It further implies that functional roles of CsGSTos might also be engaged in a relatively narrow target specific adaptive protection of specialized protein(s)/organ(s) compared to other types of GSTs, e.g. for protection of the worm’s reproductive system.

*Clonorchis sinensis* is equipped with several antioxidant enzymes to respond to and modulate exogenously- and endogenously-derived harmful byproducts. We previously reported that different species of CsGSTs might have evolved for each of the multiple specialized functions, i.e. Cs28σGST1 and its paralogues might be specialized for detoxification of endogenous toxicants, while Cs28σGST3 and Cs26μGST2 conjugate xenobiotics/hydrophobic substances in extracellular environments [9, 10]. The mu- and sigma-class CsGSTs are distributed in the tegument and parenchymal tissues [9, 41] and the phospholipid hydroperoxide glutathione peroxidases, which also revealed protective functions, are localized on the vitellocytes and eggs [25]. We assessed induction profile of CsGSTs by in vitro exposing the worms with oxidizing chemicals. CsGSTo expression increased in a dose- and a time-dependent fashion in the sperm, seminal receptacle and vitelline follicle-enriched parenchyma, but different induction patterns were evident between the two genes and between the chemicals used. CsGSTo1 showed a response relatively sensitive to Juglone compared to CsGSTo2 (Figs. 6, 7). This result might reflect greater activity of CsGSTo1 in scavenging intracellular reactive oxygen species compared to CsGSTo2, because Juglone is an internal inducer of the generation of superoxide anion from molecular oxygen during aerobic metabolism [42].

We could not demonstrate whether or not induction of CsGSTos was definitively related with their protective roles. We transformed CsGSTo expressing plasmids into *E. coli* and observed their effects during oxidative killing activity. CsGSTo overexpressing bacteria exhibited significant resistance, while control cells were vulnerable under oxidative harmful states. This result indicates a pivotal role of CsGSTos in protection of *E. coli* during oxidative killing. Molecular modeling of the active site of CsGSTos was highly comparable to those of humans (Additional file 1: Figure S1) and suggested strongly that Cys-30 (CsGSTo1) and -36 (CsGSTo2) located nearby the helix α1 might form a mixed disulfide bond with the thiol group of GSH as other GSTos do [15, 43]. Induction of CsGSTos might result in increased glutathionylation and this redox detoxification activity might be responsible for the neutralization of toxic components, thus influencing their protective activity [44].

CsGSTos expressed in eggs were not induced by oxidative stresses, but were maintained at high levels regardless of environmental conditions (Fig. 6). Eggs play critical roles for preservation and expansion of the species, therefore maintenance of cellular viability is important. However, eggs will contact an extremely hostile external environment featuring aridity/humidity, temperature fluctuation and other physicochemical toxicants when expelled from the parasite. It seems reasonable to consider that sufficient amounts of GSTos might be expressed and accumulate in the egg during intrauterine maturation.

It is noteworthy that *C. sinensis* expresses two paralogous GSTo genes, while other platyhelminths examined possess a single GSTo orthologous gene (Fig. 1). The gene is also multiplied in mammalians and insects. The duplication event appears to be a lineage- or species-specific exclusion in mammalians, in which the gene might be multiplied into paralogues during an early stage of Chordata (or mammalian) evolution. Currently we have no information regarding the selective pressure that drives the lineage-specific duplication(s) of *GSTo* genes. Whether the presence of two GSTo isotypes and biological functions specialized to these molecules are unique characteristics of *C. sinensis* or common in the Opisthorchiidae, is a question that requires further studies.

CsGSTo1 and 2 might constitute target toward development of novel chemotherapeutics by inhibiting worm’s resistance within the biliary lumen, where oxidative stress is harsh. Impediment of egg viability by control CsGSTo activity might lead to interruption of *C. sinensis* life-cycle. Therefore, CsGSTos might also be exploited as transmission blocking vaccines. It would be especially useful for management of reservoir hosts in the fields, which may ultimately contribute to control of human clonorchiasis.

## Conclusions

Our data demonstrate that functional roles of CsGSTos are specialized for protection of the reproductive system during maturation and in response to oxidative stress, thereby contributing to maintenance of parasite fecundity. CsGSTos might be involved in the cellular defense against hostile environments and/or in the target specific adaptive response to maintain cellular redox conditions. The detailed understanding on the parasitic bioactive proteins including their unique biochemical features and action mechanisms might be helpful to establish control strategies, where the parasitic diseases are prevalent.

### Addendum

During the revision process, we recognized that genes showing high sequence identity with CsGSTo1 and 2 were registered in the GenBank database under accession numbers GAA34234 and GAA51230 as glutathione S-transferase omega-1 during analysis of *C. sinensis* draft genome [35]. These genes share 92 and 98 % sequence identity with CsGSTo1 (KX163088) and 2 (KX163089) characterized in this study. Verification of actual relationships of these genes needs further elucidation.

## Abbreviations

2-DE, two-dimensional electrophoresis; 4-NBC, 4-nitrobenzyl chloride; 4-NPA, 4-nitrophenyl acetate; CDNB, 1-chloro-2,4-dinitrobenzene; CHP, cumene hydroperoxide; CsGSTo1, *Clonorchis sinensis* omega-class GST1; CsGSTo2, *Clonorchis sinensis* omega-class GST2; CsTub, Cs tubulin; DCNB, 1,2-dichloro-4-nitrobenzene; DHA, dehydroascorbate; DHAR, dehydroascorbate reductase; ECL, enhanced chemiluminescence; GSH, reduced glutathione; GST, glutathione transferase; HEDS, hydroxylethyl disulfide; Juglone, 5-hydroxy-1,4-naphthoquinone; PBS, phosphate buffered saline; qRT-PCR, quantitative real-time reverse transcription-PCR; SHG, *S*-hexylglutathione

## Additional files

Additional file 1: Figure S1. Modeling of tertiary structure of CsGSTo1 and 2. **a** The tertiary structure of CsGSTo1 and 2 was predicted with the ESyPred3D program and aligned with those of the human GSTo1 (HsGSTo1, Protein Data Bank code 1EEM) and 2 (pdb 3QAG). Boxes demonstrate the geometry of amino acid residues that constitute specific active- and the ligand binding-sites. **b** Comparison of whole three-dimensional structures of CsGSTo1 (*blue*) and CsGSTo2 (*pink*). CsGSTo1 and 2 each harbors additional amino acid stretch between α4 and α5 helices and the N-terminal extension that could not be readily determined (marked by green dotted ellipses). (TIF 2405 kb) Additional file 2: Figure S2. Expression and purification of rCsGSTo1 and 2. **a** The full-length ORFs of *CsGSTo1* and *2* were transformed into *E. coli* BL21. The recombinant proteins were induced with 0.1 mM IPTG for 4 h at 37 °C. The cells were sonicated and cleared by centrifugation. Soluble fractions were subjected to Ni-NTA affinity chromatography. His-tag was further removed by thrombin cleavage. Proteins were separated by 12 % reducing SDS-PAGE and stained with Coomassie brilliant G-250. *Abbreviations*: U, uninduced cell lysates; I, soluble fractions of the induced cells; W, washing fractions; E, purified rCsGSTo1 and 2; P, thrombin-cleaved rCsGSTo1 and 2. **b** His-tag removed rCsGSTo1 and 2 were separated by 12 % reducing SDS-PAGE, electroblotted to nitrocellulose membrane and probed with anti-rCsGSTo1 or anti-rCsGSTo2. The blots were developed with ECL. (TIF 531 kb) Additional file 3: Figure S3. Determination of optimal temperature and pH. The effects of temperature **a** and pH **b** on enzymatic activity were determined by the standard dehydroascorbate reductase assay. The reactions were initiated by adding DHA and recorded for 5 min with temperature ranges from 4 °C to 55 °C. Sodium phosphate buffer (100 mM, pH 6.2–7.8) and Tris-HCl buffer (100 mM, pH 8.0–9.4) were used to observe optimal pH. All enzyme assays were independently performed in triplicate (*n* = 3, mean ± standard deviation, SD). (TIF 102 kb) Additional file 4: Figure S4. Inhibition of dehydroascorbate reductase activity catalyzed by rCsGSTo1 and 2 with praziquantel (PZQ). Lineweaver-Burk plots showing inhibition of rCsGSTo1 and 2 activities (1/v) *versus* 1/[DHA] (mM^-1^) (**a**, **b**) or rCsGSTo1 and 2 activities (1/v) *versus* 1/[GSH] (mM^-1^) (**c**, **d**) in the absence (*diamond*) and presence of 10 μM (*rectangle*), 50 μM (*triangle*) and 100 μM (*circle*) of PZQ, with variable concentrations of DHA and GSH (0.01–100 mM). Data are plotted in double reciprocal form. Insets demonstrate determination of *K*i values. All assays were independently done in triplicate (*n* = 3, mean ± standard deviation, SD) and representative figures are shown. (TIF 207 kb) Additional file 5: Table S1. Inhibitory mode of rCsGSTo1 and 2 by inhibitors. (DOCX 18 kb) Additional file 6: Figure S5. CsGSTo1 or 2 overexpressing *E. coli* show resistance against oxidative killing activity. **a** Disc diffusion assays. LB agar media were inoculated with 5 × 10^8^ *E. coli* cells transformed with recombinant *CsGSTo* plasmids or mock vector. Filter-discs soaked with 10, 50, 100 and 200 mM concentrations of cumene hydroperoxide (CHP) were placed on the plate and incubated overnight and the inhibition zones were measured. **b** Growth curves of CsGSTo overexpressed *E. coli* BL21 under oxidative stress. The stationary-phase cultures of CsGSTo overexpressed bacteria and control cells were diluted and grown in LB broth at 37 °C until exponential phase. Aliquots were treated with different doses of CHP or Juglone (0, 0.5, 1, 2 and 4 mM) and cultured for 25 h. The growth rate was spectrophotometrically assayed every 1 h. Growth curves are representative of those from three independent experiments. *Key*: ▲, control cells; ■, CsGSTo1 overexpressing cells; ●, CsGSTo2 overexpressing cells. (TIF 1468 kb)
