# Supplementary figures and images for: Clonorchis sinensis omega-class glutathione transferases play major roles in the protection of the reproductive system during maturation and the response to oxidative stress
Source: Parasit Vectors. 2016 Jun 13;9:337. doi: 10.1186/s13071-016-1622-2 (PMC4906895; doi:10.1186/s13071-016-1622-2)

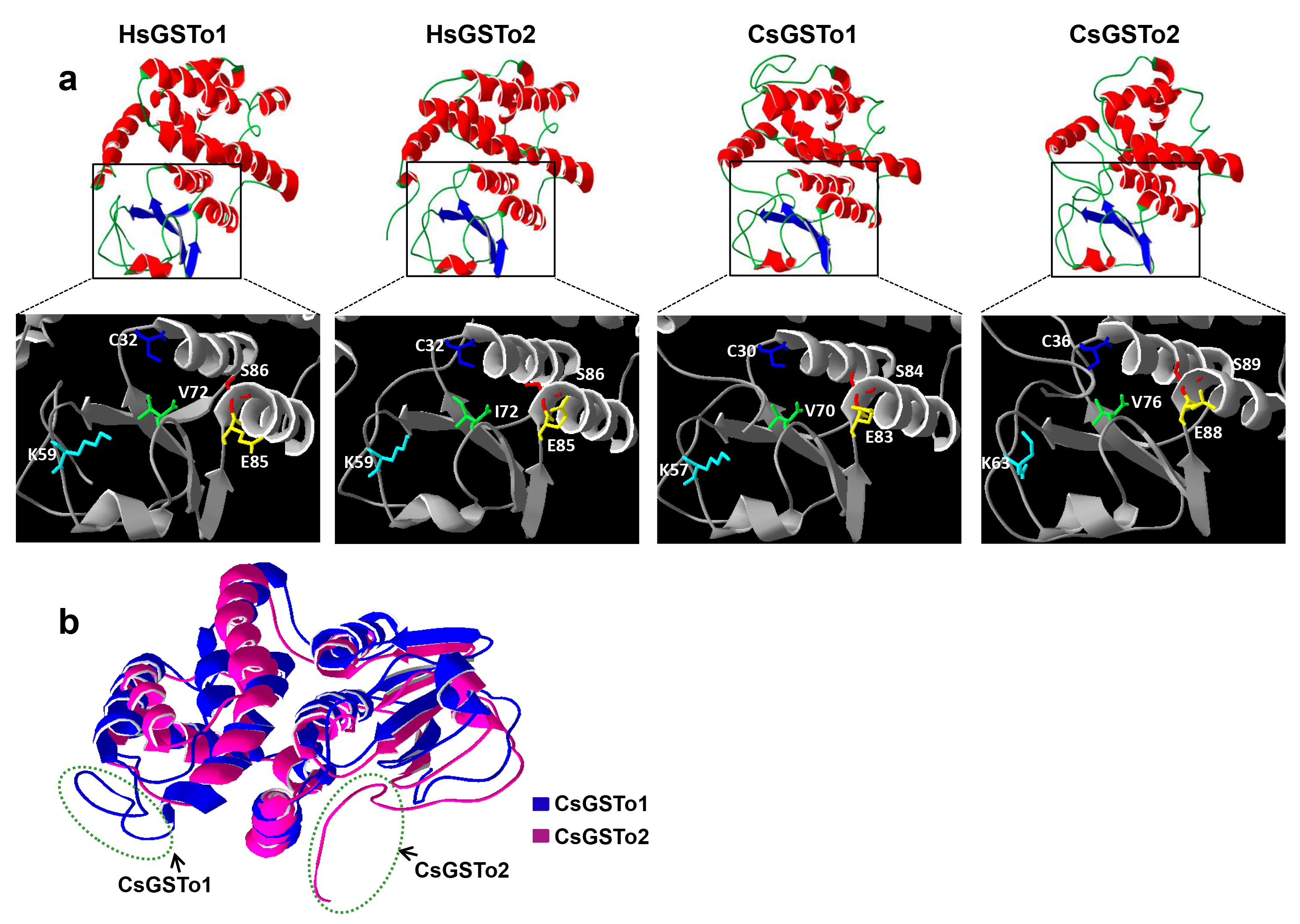

Supplement: Additional file 1: Figure S1. — Modeling of tertiary structure of CsGSTo1 and 2. a The tertiary structure of CsGSTo1 and 2 was predicted with the ESyPred3D program and aligned with those of the human GSTo1 (HsGSTo1, Protein Data Bank code 1EEM) and 2 (pdb 3QAG). Boxes demonstrate the geometry of amino acid residues that constitute specific active- and the ligand binding-sites. b Comparison of whole three-dimensional structures of CsGSTo1 (blue) and CsGSTo2 (pink). CsGSTo1 and 2 each harbors additional amino acid stretch between α4 and α5 helices and the N-terminal extension that could not be readily determined (marked by green dotted ellipses). (TIF 2405 kb) [file 13071_2016_1622_MOESM1_ESM.tif]

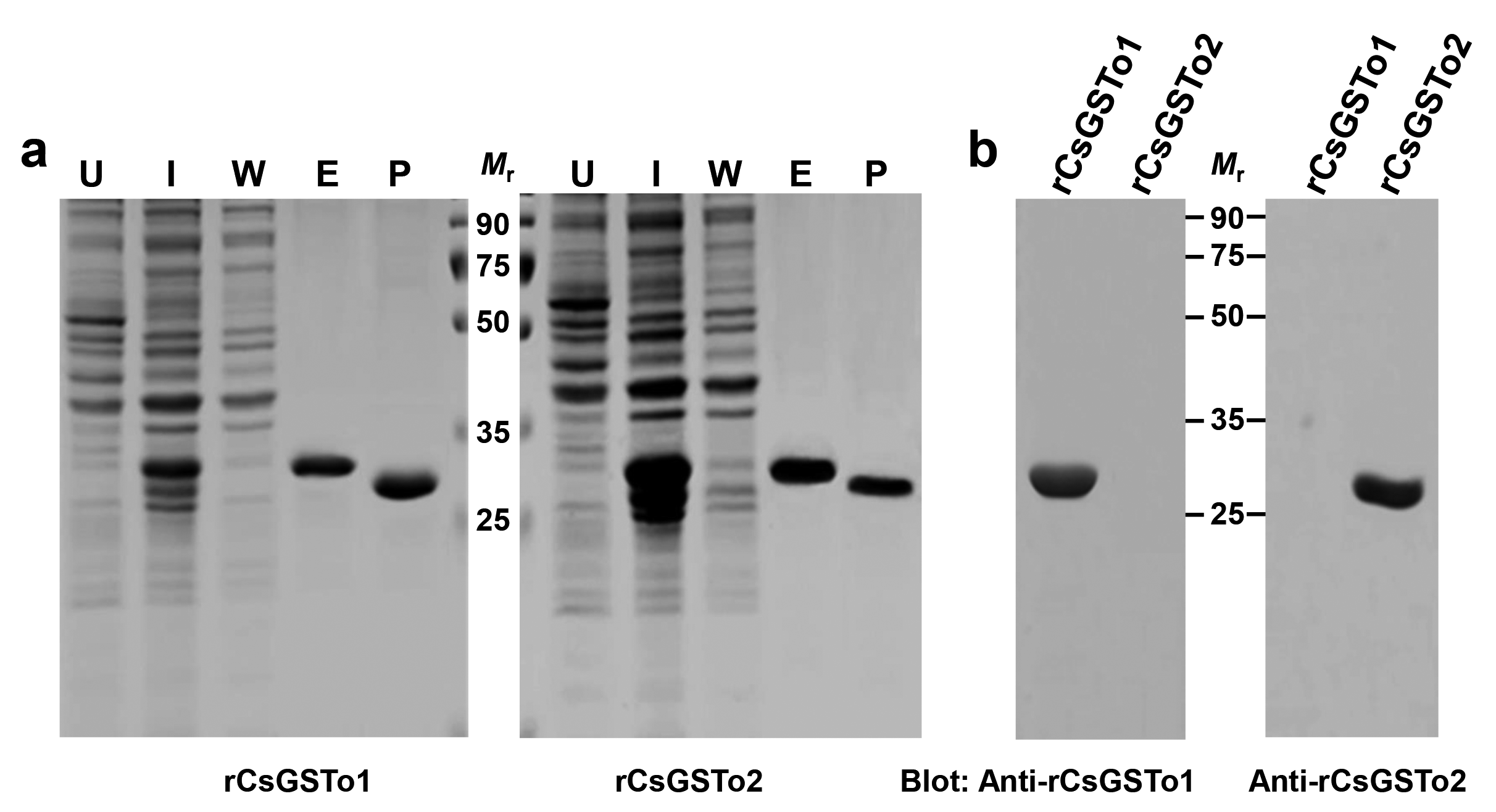

Supplement: Additional file 2: Figure S2. — Expression and purification of rCsGSTo1 and 2. a The full-length ORFs of CsGSTo1 and 2 were transformed into E. coli BL21. The recombinant proteins were induced with 0.1 mM IPTG for 4 h at 37 °C. The cells were sonicated and cleared by centrifugation. Soluble fractions were subjected to Ni-NTA affinity chromatography. His-tag was further removed by thrombin cleavage. Proteins were separated by 12 % reducing SDS-PAGE and stained with Coomassie brilliant G-250. Abbreviations: U, uninduced cell lysates; I, soluble fractions of the induced cells; W, washing fractions; E, purified rCsGSTo1 and 2; P, thrombin-cleaved rCsGSTo1 and 2. b His-tag removed rCsGSTo1 and 2 were separated by 12 % reducing SDS-PAGE, electroblotted to nitrocellulose membrane and probed with anti-rCsGSTo1 or anti-rCsGSTo2. The blots were developed with ECL. (TIF 531 kb) [file 13071_2016_1622_MOESM2_ESM.tif]

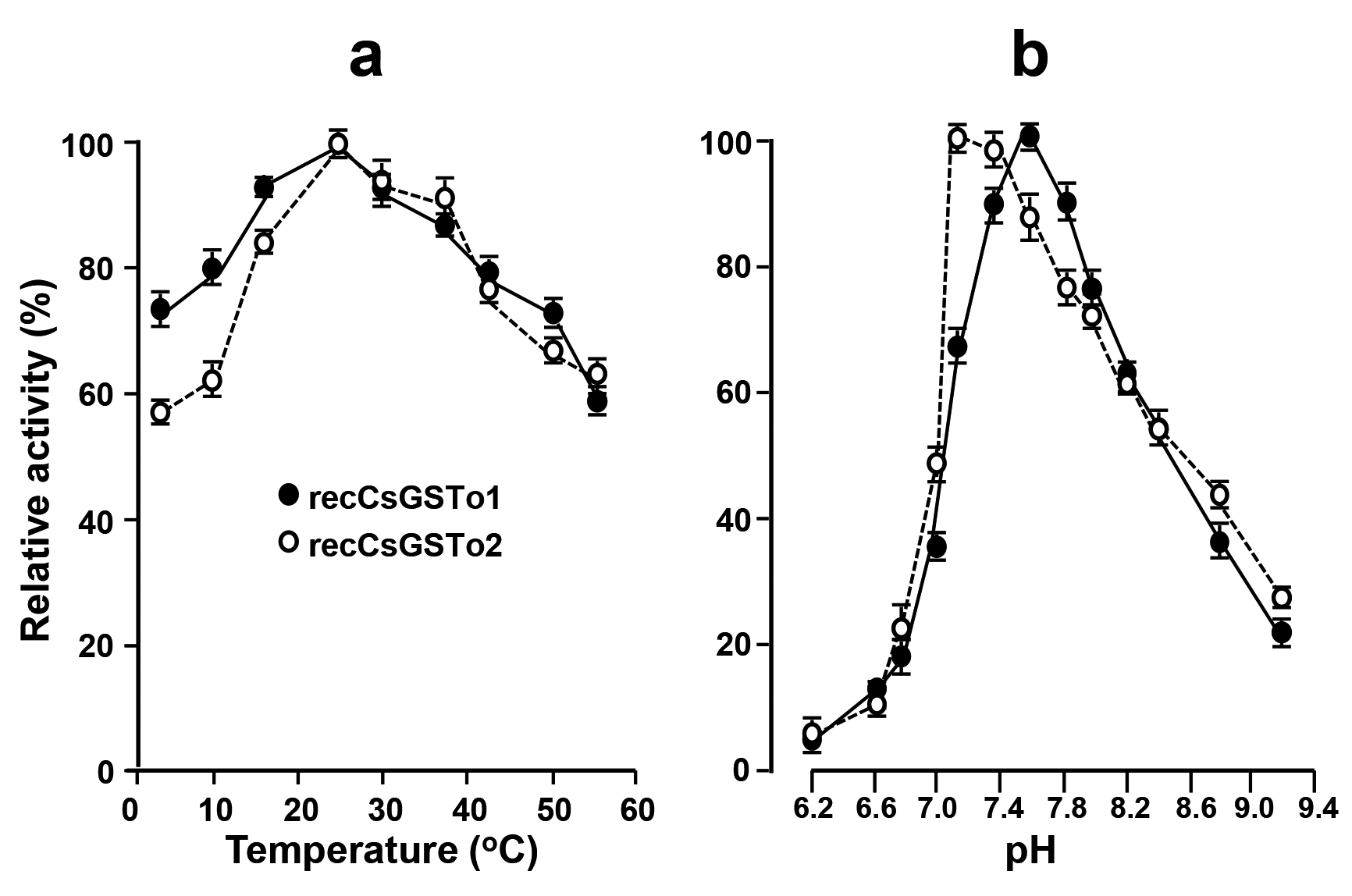

Supplement: Additional file 3: Figure S3. — Determination of optimal temperature and pH. The effects of temperature a and pH b on enzymatic activity were determined by the standard dehydroascorbate reductase assay. The reactions were initiated by adding DHA and recorded for 5 min with temperature ranges from 4 °C to 55 °C. Sodium phosphate buffer (100 mM, pH 6.2–7.8) and Tris-HCl buffer (100 mM, pH 8.0–9.4) were used to observe optimal pH. All enzyme assays were independently performed in triplicate (n = 3, mean ± standard deviation, SD). (TIF 102 kb) [file 13071_2016_1622_MOESM3_ESM.tif]

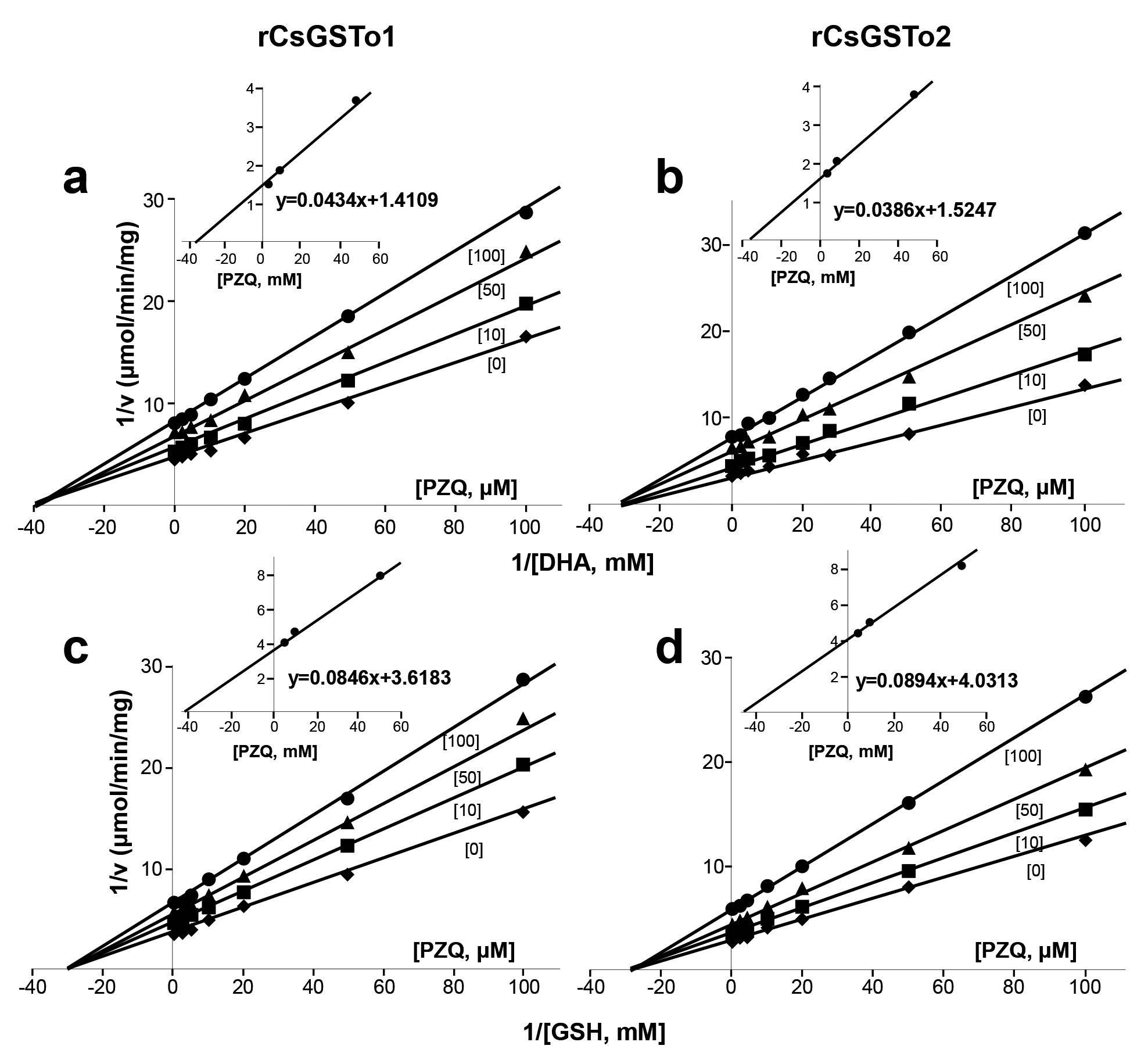

Supplement: Additional file 4: Figure S4. — Inhibition of dehydroascorbate reductase activity catalyzed by rCsGSTo1 and 2 with praziquantel (PZQ). Lineweaver-Burk plots showing inhibition of rCsGSTo1 and 2 activities (1/v) versus 1/[DHA] (mM-1) (a, b) or rCsGSTo1 and 2 activities (1/v) versus 1/[GSH] (mM-1) (c, d) in the absence (diamond) and presence of 10 μM (rectangle), 50 μM (triangle) and 100 μM (circle) of PZQ, with variable concentrations of DHA and GSH (0.01–100 mM). Data are plotted in double reciprocal form. Insets demonstrate determination of Ki values. All assays were independently done in triplicate (n = 3, mean ± standard deviation, SD) and representative figures are shown. (TIF 207 kb) [file 13071_2016_1622_MOESM4_ESM.tif]

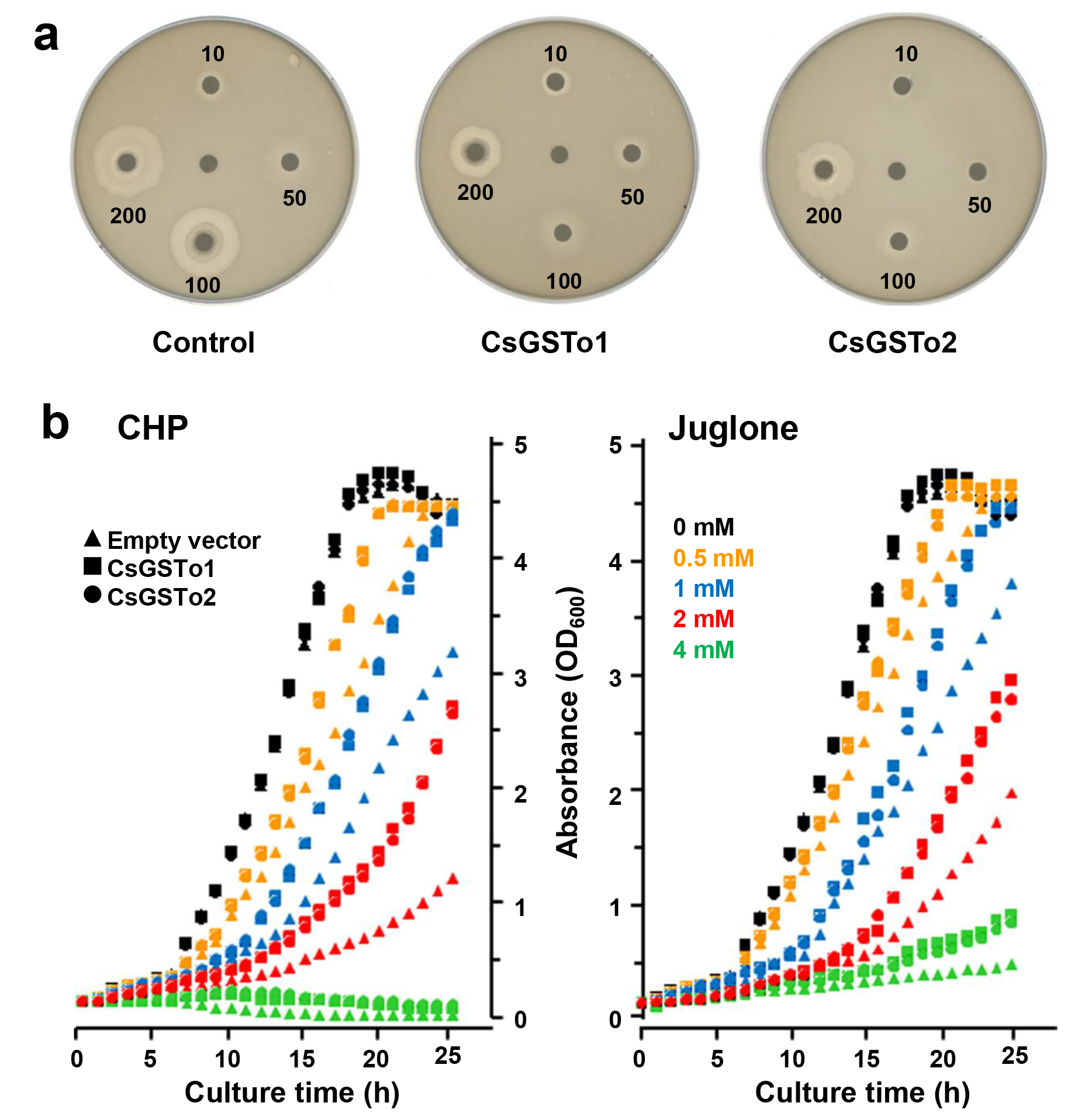

Supplement: Additional file 6: Figure S5. — CsGSTo1 or 2 overexpressing E. coli show resistance against oxidative killing activity. a Disc diffusion assays. LB agar media were inoculated with 5 × 10^8^ E. coli cells transformed with recombinant CsGSTo plasmids or mock vector. Filter-discs soaked with 10, 50, 100 and 200 mM concentrations of cumene hydroperoxide (CHP) were placed on the plate and incubated overnight and the inhibition zones were measured. b Growth curves of CsGSTo overexpressed E. coli BL21 under oxidative stress. The stationary-phase cultures of CsGSTo overexpressed bacteria and control cells were diluted and grown in LB broth at 37 °C until exponential phase. Aliquots were treated with different doses of CHP or Juglone (0, 0.5, 1, 2 and 4 mM) and cultured for 25 h. The growth rate was spectrophotometrically assayed every 1 h. Growth curves are representative of those from three independent experiments. Key: ▲, control cells; ■, CsGSTo1 overexpressing cells; ●, CsGSTo2 overexpressing cells. (TIF 1468 kb) [file 13071_2016_1622_MOESM6_ESM.tif]
