# Supplementary material for: Clonorchis sinensis omega-class glutathione transferases play major roles in the protection of the reproductive system during maturation and the response to oxidative stress
Source: Parasit Vectors. 2016 Jun 13;9:337. doi: 10.1186/s13071-016-1622-2 (PMC4906895; doi:10.1186/s13071-016-1622-2)
Supplement: Additional file 5: Table S1. — Inhibitory mode of rCsGSTo1 and 2 by inhibitors. (DOCX 18 kb) [file 13071_2016_1622_MOESM5_ESM.docx]

**Additional file 5: Table S1** Inhibitory mode of rCsGSTo1 and 2 by inhibitors^a^

| Enzymes | | Inhibitors | IC_50_SC^b^ | *K*i | Inhibition pattern^c^ |
| --- | --- | --- | --- | --- | --- |
| recCsGSTo1 | Praziquantel (PZQ) | |  |  |  |
|  | Specific active site | | 108.4 ± 0.8 µM | 44.1 µM | Non-competitive |
|  | Ligand binding site | |  | 46.8 µM | Non-competitive |
|  | *S*-hexylglutathione (SHG) | |  |  |  |
|  | Specific active site | | 0.84 ± 0.06 nM | 0.63 nM | Competitive |
|  | Ligand binding site | |  |  | Competitive |
| recCsGSTo2 | PZQ | |  |  |  |
|  | Specific active site | | 116.8 ± 0.7 µM | 38.2 µM | Non-competitive |
|  | Ligand binding site | |  | 42.8 µM | Non-competitive |
|  | SHG | |  |  |  |
|  | Specific active site | | 0.79 ± 0.04 nM | 0. 58 nM | Competitive |
|  | Ligand binding site | |  |  | Competitive |

^a^ Enzyme activity was independently assayed in triplicate and is expressed as mean ± S.D.

^b^ The half maximal inhibitory concentration values represent a saturating concentration

^c^ Non-competitive inhibition is a type of inhibition mode that involves an inhibitor bound near the substrate binding site, thereby affecting the topology of the binding pocket.
